# Supplementary figures and images for: Characterizing Emerging Canine H3 Influenza Viruses
Source: PLoS Pathog. 2020 Apr 14;16(4):e1008409. doi: 10.1371/journal.ppat.1008409 (PMC7182277; doi:10.1371/journal.ppat.1008409)

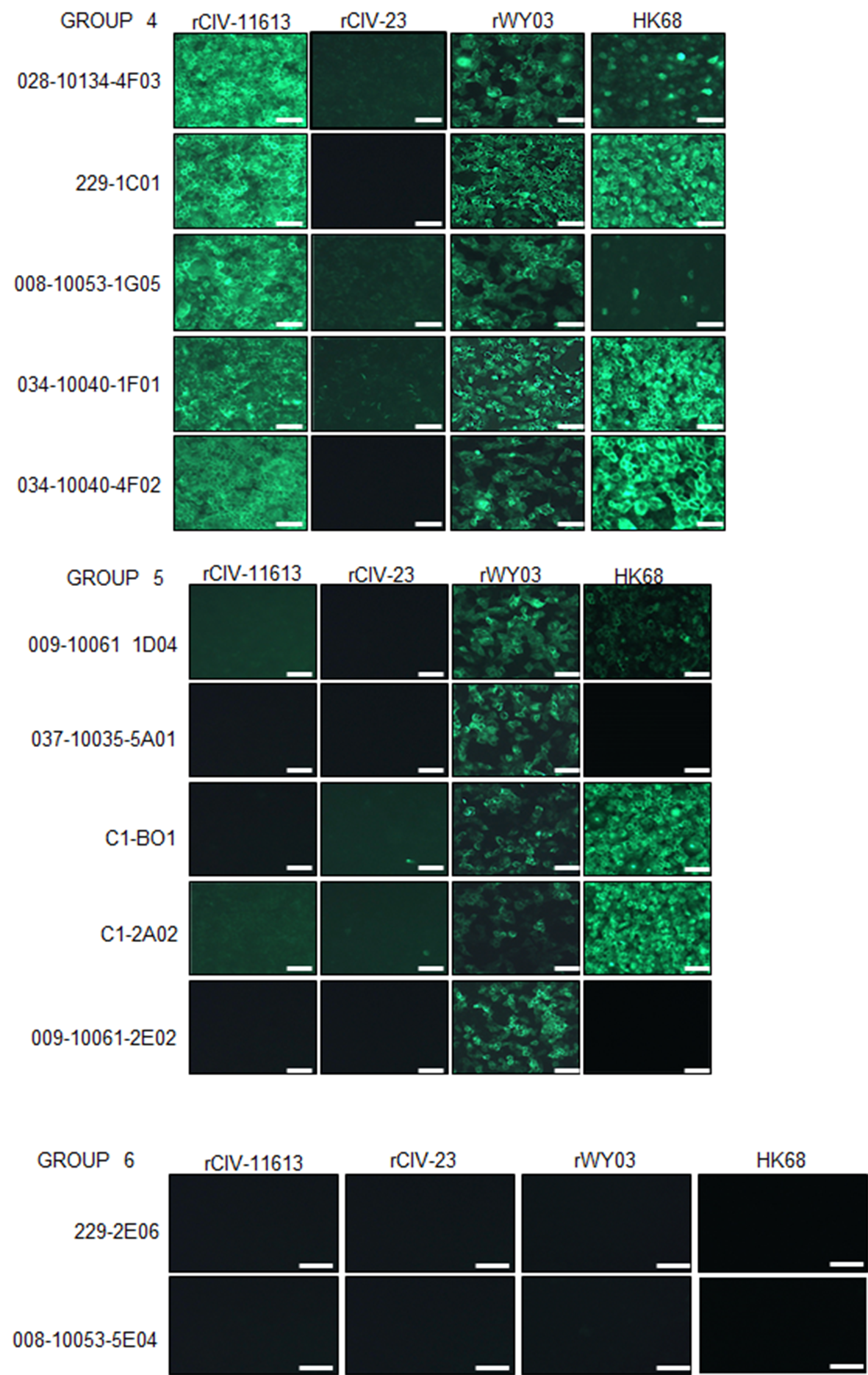

Supplement: S1 Fig — MDCK cells were infected (MOI of 3) with H3N2 or H3N8 rCIV, rWy03 H3N2 and HK68 H3N2. At 12 hpi cells were fixed, permeabilized and incubated with 1 μg/ml of the indicated hmAbs. After incubation with a secondary anti-human FITC-conjugated Ab, fluorescence was imaged under a fluorescent microscope. The hmAbs were grouped based on their ability to recognize H3N2 and H3N8 rCIV, and rWy03 H3N2. Representative images of the reactivity of the hmAbs against infected cells are shown in the right. The scale represents % of recognition. Scale bars, 200 𝜇m. (TIF) [file ppat.1008409.s001.tif]
