# Supplementary material for: Characterizing Emerging Canine H3 Influenza Viruses
Source: PLoS Pathog. 2020 Apr 14;16(4):e1008409. doi: 10.1371/journal.ppat.1008409 (PMC7182277; doi:10.1371/journal.ppat.1008409)
Supplement: S1 Table — Sera was tested for HAI titers against select H3 CIVs. (DOCX) [file ppat.1008409.s002.docx]

|  | | |  |
| --- | --- | --- | --- |
| S1 Table. Human HAI titers against H3 CIV. | | | |
| Age range (years) | # positive/total tested (% seropositive) HAI antibodies > 1:40 | | |
|  | Guangzhou^a^ | Santa Marta, Colombia^b^ | |
| 0-10 | 0/25 (0%) | 0/16 (0%) | |
| 11-20 | 2/25 (8%) | 0/10 (0%) | |
| 21-30 | 0/25 (0%) | 0/6 (0%) | |
| 31-40 | 1/25 (4% | 0/9 (0%) | |
| 41-50 | 0/25 (0%) | 0/8 (0%) | |
| 51-60 | 1/25 (4%) | 0/2 (0%) | |
| 61-70 | 3/25 (12%) | 0/2 (0%) | |
| 71-80 | 5/25 (20%) | NA^c^ | |
| >80 | 6/25 (30%) | NA^c^ | |
| ^a^Tested against rCIV-1177  ^b^Tested against rCIV-1177and CIV-41915  ^c^Not available | | |  |
