# Supplementary material for: Characterizing Emerging Canine H3 Influenza Viruses
Source: PLoS Pathog. 2020 Apr 14;16(4):e1008409. doi: 10.1371/journal.ppat.1008409 (PMC7182277; doi:10.1371/journal.ppat.1008409)
Supplement: Attachment — Submitted filename: CIV manuscript response to reviewers final.docx [file ppat.1008409.s003.docx]

Dear Editor and reviewers,

Thank you for your positive reply and thoughtful comments on our manuscript “Characterizing Emerging Canine H3 Influenza Viruses” that we have submitted to PLOS Pathogens.

We have carefully considered all the reviewers’ comments and suggestions, which have helped us to improve the quality of our manuscript. To address the comments made by the reviewers, as well as to accommodate their suggestions, we have introduced several changes highlighted in gray in the new version of the manuscript. The following is a point-by-point response to the comments raised by each one of the reviewers.

**Part I - Summary**

**Reviewer #1:** This paper describes the efforts of a large consortium of CEIRS-funded investigators to perform quantitative risk assessment on the potential for human emergence of recently circulating canine H3 viruses. This is meant as a proof-of-principle of the utility of the CEIRS network for supporting large, multi-investigator efforts aimed at facing specific public health threats posed by influenza viruses. They describe a comprehensive assessment of phenotypic correlates of emergence potential for a small sample of canine H3N2 and H3N8 viruses. They show variable ability of canine viruses to replicate in primary human cells from upper vs. lower airway, that they can readily transmit via contact between guinea pigs, receptor binding profiles and pH characteristics of HA, that the viruses are sensitive to licensed antivirals, and that people born after 1970 have limited immune recognition of canine H3s. Plus a bunch of other stuff that I don’t need to list. Altogether, they conclude that canine H3 viruses pose a very limited emergence threat and suggest that they would be effectively controlled by existing antivirals and broadly-reactive anti-H3 therapeutic monoclonal Abs.

Overall, the paper presents a nice, comprehensive data set that addresses an important open question. With a couple of minor exceptions, the conclusions are well supported by the data, and the experimental approaches used are sound. Given the huge number of labs involved, the paper is well-written and cohesive, but is a little sloppy with some missing or unexplained figures and poor descriptions of some of the data/rationales. It also comes across as weirdly sales-pitchy in terms of the CEIRS angle. The paper would benefit from toning that down a bit and focusing on the science.

**Response: We thank the reviewer for the positive comments. We have thoroughly revised the manuscript according to her/his suggestions.**

**Reviewer #2:** This is an unusual manuscript to be asked to review since its prime claim seems to be whether the NIAID CEIRS collaboration was able to provide research results that can be used to perform a risk assessment of the pandemic potential of influenza viruses from dogs. The risk assessment frameworks for the results to be applied to are the Influenza Risk Assessment Tool (IRAT), developed by CDC, and the Tool for Influenza Pandemic Risk Assessment (TIPRA), developed by WHO. The unusual question that has been posed is reflected in the unusual type of title of the manuscript.

**Response: We concur with the reviewer and have modified the manuscript accordingly.**

**Reviewer #3:** In the research article entitled “Characterizing Emerging Canine H3 Influenza Viruses: Challenging the Centers of Excellence for Influenza Research and Surveillance (CEIRS) Network Pipeline”, the authors test the efficiency of the CEIRS network to respond to emerging threats. It is critical to perform these exercises to ensure productive cross-network interactions and assess response times. Importantly, the authors identify key areas, based on this exercise, that can be improved upon for future studies with emerging pandemic threats. This assessment is not only useful for the CEIRS network but also for other scientific centers tasked with addressing large research areas.

In addition to testing the CEIRS network response capability, the authors provide a wealth of data on emerging canine influenza viruses (CIV) as a pandemic threat. In this area the authors test the transmissibility of CIV in multiple animal models, characterize the phenotype of these viruses, and assess pre-existing immunity in the human population by age. Below are specific comments for each of the parameters tested of CIV that would assist the reader in following this comprehensive study.

**Response: We thank the reviewer for the positive comments.**

**Part II – Major Issues: Key Experiments Required for Acceptance**

**Reviewer #1:**

- Many of the experimental efforts did not include a representative H3N8 virus. Is there a reason for that? Without this, it is impossible to determine how broadly the results in question extend to all canine H3 viruses or just H3N2s.

**Response: The H3N8 viruses were used in the human immunological characterization studies only to assess more broadly reactive H3 responses. This is not stated on line 82 and justified on line 112.**

- Figure 12 is missing

**Response: Apologies for the oversight. It is now included as Figure 11.**

- The ferret transmission experiment uses a physiologically irrelevant infection dose of 10^6 pfu. I recognize that the flu field has settled on this dose as the gold standard for ferret infections for some reason, but it makes it impossible to determine whether the transmission results in that experiment reflect what would be expected during natural infection. This experiment should either be repeated using a relevant dose (as was done for the guinea pig experiments), removed form the paper, or the text should clearly highlight that the results may be non-representative of reality due to the experimental design.

**Response: We appreciate the reviewer’s concern and now include the caveat that these studies many be non-representative of the situation in nature on lines 188 – 189.**

**Reviewer #2:**

- The panel of viruses examined included natural H3N2 viruses from dogs and their RG equivalents, an H3N8 virus from dogs and two RGs based on it, along with a panel of human H3N2 viruses and some RG reassortants. The passage histories of the viruses have not been given and, for human H3N2 viruses at least, passage history and the changes acquired on passage need to be taken into serious consideration. Whether this is the case for canine H3 viruses ought also to be considered.

**Response: The passage histories of the viruses are now included.**

- Table 3 shows the low pH inactivation of virus and the pH of syncytium formation by three viruses. The authors need to present a conclusion on whether the differences observed between the pH of inactivation of the two canine viruses is significantly different from the single example of a human H3N2 virus. The conclusion presented on lines 193-194 seems to require further justification.

**Response: We agree. This section has been rewritten.**

- The authors describe pathogenicity studies in mice and show that the DBA/2J mice are sensitive to infection by CIV-41915 and rCIV-1177. It might be worth explaining whether CIV-41915 and rCIV-1177 resulted in the death of those mice scored as fatally infected or whether they were culled having reached a humane end-point. It Is not clear what conclusion ought to be reached with such varying results between inbred mouse lines.

**Response: We thank the reviewer for their comment. Mortality of DBA/2J mice inoculated with CIVs resulted in a combination fatal infection, and euthanasia upon reaching humane end points. The increased mortality of the DBA/2J strain following influenza A virus infection is consistent with previously published studies demonstrating that DBA/2J mice are more susceptible to influenza A virus infection, than the influenza resistant mouse strain, BALB/c1,2. We have now discussed this aspect within the section, Pathogenicity in mice. Some references supporting this include:**

1 Boon, A. C. M. et al. H5N1 Influenza Virus Pathogenesis in Genetically Diverse Mice Is Mediated at the Level of Viral Load. mBio 2, e00171-00111, doi:10.1128/mBio.00171-11 (2011).
2 Pica, N. et al. The DBA.2 Mouse Is Susceptible to Disease following Infection with a Broad, but Limited, Range of Influenza A and B Viruses. J. Virol. 85, 12825-12829, doi:10.1128/jvi.05930-11 (2011).

- Figure 6 shows the results of transmission between infected ferrets or guinea pigs and direct contacts. The style of the results being presented ought to be generally similar. Figure 5A does not seem to look to be data from nine single animals (as described in the materials and methods (lines 633 to 634) and here it is implied that more than one virus was tested for ferrets).

**Response: Apologies for the confusion. Experiments used a total of 9 ferrets with n=3 per group; donor, direct contact, and respiratory contact. This is now detailed on line 571.**

- Table 5 and Figure 6 show antiviral sensitivity results. Here different viruses were assessed by different methods. It is particularly striking that the absolute sensitivity of the different viruses to the drugs are different in the different assays. This is not perhaps surprising for the NA inhibitors (a sialidase inhibition assay versus a virus replication assay) but the assays for amantadine resistance have similar types of read-outs and the sensitivity of the viruses to amantadine appear to be very different indeed. That said, there is no doubt that the conclusions drawn overall are robust.

**Response: We agree. Per reviewer’s comment and to avoid confusion, we kept in the revised manuscript the data that were obtained in accordance with WHO and CDC recommendations (Table 5) and deleted the data that were obtained using recombinant mCherry-expressing viruses.**

- Antigenic differences between the viruses and circulating human viruses have been assessed in several ways. Amino acid sequence-based methods have been presented here extensively based on the claim (lines 246 to 248) that these methods can predict antigenic relatedness. Figure 7 shows a cluster analysis of the viruses but this does not directly assess the antigenic relatedness of the viruses within a subtype. No assessment of antigenic discrimination by post-infection ferret antisera has been given – yet the sera might well have been collected in the studies. With these sera the hypotheses presented in the paragraph from lines 271 to 280 (particularly lines 278 to 280) about the relatedness of the CIV viruses to old H3N2 viruses ought to have been tested directly.

**Response: There is currently no ferret antisera to the canine viruses, which is why they were not mapped that way. Virus genome data can be used to compare primary protein structures between HA and NA antigens [32-24 and Sci Rep 2017;7:14614.] which have been shown to correlate with antigenic changes [34 and Miller paper]. SBM gives us a ferret-free alternative that can be used to more rapidly assess antigenic relatedness. Both canine H3 HA are most closely related to other avian or equine-origin H3 HAs, as demonstrated in Figure 7. The cN2 NA clusters with avian N2 strains, also having emerged from birds, and the N8 cNA clusters with equine NAs , and we know those were passed to dogs from horses. We have revised the text for clarity.**

- Human immunity has been subsequently analysed. It is very surprising that there are such high titres seen to the A/Wyoming/3/2003 antigen by HI. I would presume that these sera would be considered to be the equivalent of pre-vaccination sera and a typical panel of pre-vaccination human sera might have mean GMTs of 30 to 100. These HI titres presented here for A/Wyoming/3/2003 look very much higher with some antisera having titres of over 1,000 and so the protocols used need to be reassessed by the panel of authors. With the HI panel showing such a concern for A/Wyoming/3/2003, all the other results also need to be carefully reassessed.

**Response: We were not surprised to see high HAI titers to WY/03. This has been observed in other studies (Clinical Infectious Diseases 2019;68(12):2067–78 and Clinical and vaccine immunology: CVI 2011;18:469-476) and it was chosen for its high reactivity. We also see high titers to A/Texas/2012 H3N2 using the same sera (see below).**

- Human post-vaccination sera were also analysed with two panels of sera. The authors describe a ‘baseline’ titre of neutralising antibody – I have presumed that this is pre-vaccination titres. It is a pity that these were not also assessed by HI assay as well as the VN assay. The results shown in figures 8B and 8C seem to show pre-vaccination and post-vaccination titres in the figure legend, but this is not what is shown in the figure which shows unvaccinated and vaccinated individuals. I was confused which was the correct interpretation. Pre- and post-vaccination paired sera would present the strongest evidence for any cross-reactivity or not.

**Response: We apologize for the confusion. Baseline represents the NAb at the time of enrollment. For figures B and C, sera was collected at the time of hospital confirmation of H3N2 infection and vaccination status during the 2016-2017 season was documented. The individual responses are then clustered based on vaccine status. We agree that testing pre and post-vaccination paired sera would be ideal, but that was unavailable for these studies.**

- Human mAbs were also analysed. Here a large panel of mAbs have been put into groups but the epitopes recognised by the panel of mAbs do not seem to have been defined and this makes interpretation of the results difficult. This also applies to Figure 11 – and the coding had to be deduced from the results. These results were also very difficult to interpret. Table 6 is not well described or well presented. The significance of the results in this whole section is difficult to establish.

**Response: We agree and have modified these figures and revised the manuscript accordingly. While the mAbs have been characterized by Dr. Wilson’s group and used in similar studies (PMID: 29985597), information on the epitopes targeted by the mAbs is not currently available.**

- One of the key elements of WHO’s TIPRA is for the experts to make an assessment of the confidence scores for each of the scoring elements. Here this does not seem to have been carried out; this could well have been done either in the results section or, perhaps better, in the discussion. This assessment would allow the authors to assess the significance of their own results. An important point of the risk assessment frameworks is to assess where there is limited information, thereby indicating what types of new data are required for improved risk assessments. The authors should take an opportunity to do this. They might cover the points raised here about difficulty in interpretation of some of the presented results.

**Response: We agree but were unable to gather the required experts within the timeframe for a proper analysis. Thus, we toned the language throughout the manuscript on the data being used to inform the algorithms. However, it would be interesting to determine how/if our data impacts previous risk assessments on the CIVs made by other groups.**

- The discussion, as it stands, repeats many of the points covered in the introduction. However, the discussion raises other very important points – particularly relating to the use of RG viruses. The authors offer a possible solution to this.

**Response: We agree. The discussion has been revised.**

**Reviewer #3:**

- No Response

**Part III – Minor Issues: Editorial and Data Presentation Modifications**

**Reviewer #1:**

- Rationale for the specific virus strain selections in fig 1 and fig 2 not well explained

**Response: We agree. This is now explained on lines 103 – 115.**

- Line 158: data do not actually show a comparison between natural and recombinant versions of 11613, the natural isolate tested is 41915.

**Response: This figure has been removed.**

- Fig 2: no description of 2E anywhere, and 2D is not described in the legend

**Response: Apologies. This has been corrected.**

- Fig 3: x-axes would help. Also, the examination of a single canine isolate seems insufficient to draw broad conclusions. should compare representatives from H2N8 and H3N2 trees

**Response: A new figure has been included.**

- Line 279: Does this analysis provide any information not provided by a maximum likelihood phylogenetic tree of the relevant gene segments?

**Response: This analysis explicitly examines the changes in sequences that fall within structures of the HA in the regions associated with antibody recognition. As such the goal is to estimate the likely antigenic distance between viruses, rather than the just the genetic evolutionary distances which would be seen in a more general phylogenetic analysis of the HA sequences. While those two types of analysis may sometimes give similar conclusions, the cartography analysis, by focusing on the antigenic structure, is more likely to reflect differences in immune (antibody) cross-protection."**

**Some references to the specific methods include:**

[**https://www.ncbi.nlm.nih.gov/pubmed/29433425**](https://nam03.safelinks.protection.outlook.com/?url=https%3A%2F%2Fwww.ncbi.nlm.nih.gov%2Fpubmed%2F29433425&data=01%7C01%7CStacey.Schultz-Cherry%40STJUDE.ORG%7C27242b841eb74447123e08d7769ebb26%7C22340fa892264871b677d3b3e377af72%7C0&sdata=JmrfyP%2FxDzLCWVSTZrAm0t3hzx9oz5A29mtYU8p%2FMhU%3D&reserved=0)

[**https://www.ncbi.nlm.nih.gov/pubmed/28098392**](https://nam03.safelinks.protection.outlook.com/?url=https%3A%2F%2Fwww.ncbi.nlm.nih.gov%2Fpubmed%2F28098392&data=01%7C01%7CStacey.Schultz-Cherry%40STJUDE.ORG%7C27242b841eb74447123e08d7769ebb26%7C22340fa892264871b677d3b3e377af72%7C0&sdata=kkudHdBe02Yzx8dibQyXXs1DWjPUcX%2F%2BEU55nvJLzMQ%3D&reserved=0)

- Figure 7: For consistency, would be good to indicate the specific strains used in the human serological analyses in figure 8 on these plots.

**Response: We agree with the reviewer have indicated the positions on the revised figure.**

- The high, age-independent ELISA and ELLA titers against the canine N2 and N8 proteins are surprising, as the authors note. Does this suggest that the sequence-based antigenic analysis fails to accurately capture something important about NA-Ab interactions?

**Response: We believe the clustering data in Figure 7 supports the NAI (ELLA) results in Figure 8. The canine N2 clustered close to several human N2 strains, which exposure to would contribute to the reactivity observed in the NAI. Similarly, the N8 NA did not cluster near any human viruses, suggesting minimal exposure to N8-like NA in humans and accounting for low NAI titers. As for the ELISA results, the SBM clustering using full HA sequence is primarily driven by the differences in the HA head domain given its relatively much greater diversity compared to the stalk domains. When using only stalk derived sequence data, SBM can resolve differences (see our Sci Reports MS) The ELISA reactivity in Fig 8A may reflect some stalk immunity.**

- Figure 10E: legend is insufficient. What are values shown on heat map? Also, do the IF data suggest that AIR may miss some cross-reactivity? For example, 017-10116-5B03 shows maximal signal against rWY03 by IF but looks like it has no signal against this virus by AIR? It looks like the discordance between the two datasets is more complicated than simply a greater degree of sensitivity of AIR vs. IF as the authors claim. For example, 028-10134-4F03 apparently shows strong reactivity with rCIV-11613, moderate reactivity with rWY03 and little to no reactivity with rCIV-23 and HK68 by IF in figure 11, but the same mAb shows significant reactivity with the two canine IAVs but not HK68 or rWY03 by AIR in fig. 10. This is a qualitative difference in the predicted reactivity, not simply a difference in sensitivity. There may be others as well, but I don’t have time to scan through the whole thing. The authors should confirm whether the differences in results provided by the two methods are a consistent sensitivity issue or are more complicated.

**Response: Apologies. We have modified the legend to provide additional information regarding the array images and heat map. Regarding the reviewer’s question about antibody 017-10116-5B03 and rWY03, this antibody was not on the AIR array, and we did not examine the response of the array with rWY03.**

**Reviewer #2:**

The manuscript has the signs of being written by several authors. I would suggest a single author goes through the whole manuscript (particularly in the materials and methods section) to ensure a consistent standard of use of English and the amount of detail given.

**Response: Thank you for the comment. The manuscript has been revised for accuracy and consistency.**

Line 31. The words ‘minimal risk’ are probably not the appropriate words to describe the outcome since ‘low risk’ would be better. It is easy to think of influenza viruses of lower risk to humans than the mammalian adapted canine influenza viruses.

**Response: We agree and have modified accordingly.**

Line 90. If the authors mean Low Pathogenicity (or Low Pathogenic) Avian Influenza viruses then this should be clearly stated as it has a precise meaning in OIE regulations and other legislation, whereas nonpathogenic is not well defined.

**Response: We agree with the reviewer and we have modified the document.**

Line 96. It would be nice to provide a few more words about the nature of the human influenza viruses isolated from dogs and how well they were characterised.

**Response: This information is now included in lines 57-59.**

Lines 201 and 211. PFU units were used for the infection of guinea pigs and TCID50 units were used for infecting ferrets. It would be good if the authors were able to equate the infecting doses.

**Response: While we agree, we would be hesitant to guestimate and would need to run the experiments with the exact viruses used by the different labs. Unfortunately, this was not done.**

Line 205. To reduce the possibility of confusion, I think the term ‘line’ should be used for the mice rather than the word ‘strain’, which might be taken to refer to a virus.

**Response: Modified.**

Lines 278 and 321 and Table 2 – I suspect the authors might be referring to A/Hong Kong/4801/2014 since this was presumably the vaccine virus used (line 321) rather than A/Hong Kong/1/2014.

**Response: The reviewer is correct. Apologies for the mistake.**

Line 537. A reference to TEC MM is needed.

**Response: This information has been included.**

Line 540. I suspect the word ‘residue’ might well ought to have been ‘residual’.

**Response: Thank you for pointing that out. Corrected.**

Lines 592-593, the nature of the MDCK Luc9.1 cell needs to be given.

**Response: This information is now included.**

**Reviewer #3:**

Addition of a timeline figure to visually demonstrate the length each component took the contribution of each CEIRS centers and highlight areas where the networks worked together would be very useful for this exercise. Based on the discussion (line 430-431) it seems that all of this work was completed within 2 months – which is outstanding for a first test and highlights the power of the CEIRS network.

**Response: While we appreciate the reviewer’s comment, we chose not to include a timeline to avoid “ruffling feathers”. Numerous groups were assigned parallel responsibilities and of course that means one group “wins” by completing the task first. For example, several groups were tasked with making the reverse genetics viruses due to differing methods. Yoshi’s group won. Hands down. Yet several other groups provided viruses after Yoshi. We would not want to highlight that in a timeline. We hope the reviewer can appreciate our dilemma.**

List viruses used in study
• Please add the passage history for both natural isolates and recombinant viruses to this list, as it may be distinct for each virus and important to understand if they were passaged in eggs prior to analysis for viral phenotypic characteristics. Especially since these details are not provided in the materials and methods section.

**Response: This information is included in the revised manuscript.**

Replication in primary human respiratory epithelial cells and explants:
• CIV-1177 is denoted at recombinant in the text (line 163) but not in figure 2 or line 168. Given that both recombinant and non-recombinant strains were used, it is necessary to ensure that each is denoted accurately for the reader.

**Response: We agree and have corrected the manuscript.**

Transmission in ferrets and Guinea pigs
• Line 216-217 states there was ‘no airborne transmission’ of CIV-41915 yet one animal shed detectable virus on day 3 post infection. Could the authors please provide the serology for this airborne transmission study as well as the one for rCIV-1177 which is listed as data not shown.

**Response: Thank you for pointing this out. A revised figure has been included and the section has been modified accordingly to include the HAI titer (Lines 184-189).**

Cross-reactive human and CIV antibodies
1. Figure 8 and 9 are very well done and present exciting observation critical to address pandemic emergence and the proportion of the population that is susceptible to CIV.

**Response: Thank you.**

2. The use of AIR is important – but the data presented in figure 10 is difficult to interrupt for the general reader. The majority of the antibodies tested are not specific to any virus and overwhelm part E. In addition it is unclear how A-D relate to the antibodies presented in figure E. Please adjust this presentation of these data to make it more reader-friendly. Also, a different nomenclature for the antibodies might be useful. A minor note is that some rows do not line up on the left edge of the heatmap – which is surprising and this reviewer is left wondering whether the heatmaps were generated at multiple times and then spliced and fit together? It seems as though all the data should be imported into the same script for heatmap generation.

**Response: We agree and have modified the figures and the text to improve clarity. Specifically, the array map (previous Fig S3) is now included as Fig 8, we have clarified that the array images in Fig 9A-D are the raw data used in a very sensitive chip reader to assess relative reactivities, and have modified Figure 9 to make it more reader friendly by indicating the MAbs used in Figure 10.**

**Regarding the nomenclature of the antibodies, we would like to keep the nomenclature used in our initial submission of the manuscript to be consistent with that published in our prior published work (2018 Analytical Chemistry) and documented in Patrick Wilson’s lab, from which the antibodies MAbs originate.**

3. Could the authors please denote them in figure 10 so the reader can easily compare the AIR profile to the cross-reactive IF profile. Even though it is presented on Table 6 – it would be nice to assess it in the figures. On Table 6 – could the authors add dividing lines between groups and antibodies.

**Response: We agree and have included in new Figure 9 (AIR assay) the antibodies and groups listed in new Figure 10.**

In vitro neutralization of H3 CIVs with hmMAbs
• Figure 12 was not included in the manuscript so nothing could be assessed. Although the NT titers are provided in Table 6 – suggesting that Figure 12 is unnecessary.

**Response: Apologies. Fig 12 is now Fig 11 and has been included.**

A final comment is that there are a number of grammatical typos that should be fixed. Some examples include lines 94 (their -> the), 106 (add in ‘to’ between obtained and inform), 348 (30 should come after ‘of the’).

**Response: Apologies. Manuscript has been revised accordingly.**
