# Supplementary material for: Characterizing Emerging Canine H3 Influenza Viruses
Source: PLoS Pathog. 2020 Apr 14;16(4):e1008409. doi: 10.1371/journal.ppat.1008409 (PMC7182277; doi:10.1371/journal.ppat.1008409)
Supplement: Attachment — Submitted filename: CIV manuscript response to reviewers.docx [file ppat.1008409.s004.docx]

Dear Editors,

Thank you for your positive reply and the acceptance of our manuscript “Characterizing Emerging Canine H3 Influenza Viruses” in PLOS Pathogens. We have considered all the editorial suggestions and we have introduced the requested changes in the new version of the manuscript. The following is a point-by-point response to these editorial comments.

**Comment 1.** The phrase "the CEIRS network could work together..." is repeated at least three times in the first few pages of the manuscript (in the abstract, last paragraph of the intro, and first paragraph of the results section). Please remove this sentence from the abstract and introduction as it distracts from the scientific rationale of the work.

***Response:*** *We have removed the sentence “the CEIRS network could work together…” in the abstract and introduction of the revised manuscript.*

**Comment 2.** In the abstract, please either expand on the "valuable lessons learned" (last sentence) or remove this sentence from the abstract as it is vague.

***Response:*** *We have removed this sentence in the abstract of the revised document.*

**Comment 3.** Unites States is define as US in line 45, but this definition is not used in the remaining of the manuscript.

***Response:*** *We apologize for this editorial mistake that have been fixed in the revised manuscript.*

**Comment 4.** line 58: A/Hong/Kong... please add influenza A virus.

***Response:*** *We apologize for this mistake that have been fixed in the revised document.*

**Comment 5.** line 78: "preexisting antibody (Ab) immunity"...that wording is not conventional use preexisting antibodies or preexisting humoral immunity.

***Response:*** *This sentence has been changed in the revised document.*

We hope that by taking into account these editorial comments and suggestions, our manuscript would be now accepted for publication at PLOS Pathogens. Finally, we want to thank again the reviewers and editors for their helpful and constructive comments on our manuscript.
